# Progress in the study of biological effects of hydrogen on higher plants and its promising application in agriculture

**DOI:** 10.1186/2045-9912-4-15

**Published:** 2014-08-20

**Authors:** Jiqing Zeng, Zhouheng Ye, Xuejun Sun

**Affiliations:** 1 Key Laboratory of South China Agricultural Plant Genetics and Breeding, Key Laboratory of Plant Resources Conservation and Sustainable Utilization, South China Botanical Garden, Chinese Academy of Sciences, Guangzhou, China; 2 Department of Navy Aeromedicine, Second Military Medical University, Shanghai, China

## Abstract

While the medical effects of hydrogen have been broadly analyzed, research into the effects of hydrogen on higher plants has often been of lesser concern. Recent studies on the botanical effects of hydrogen have shown that it is involved in signal transduction pathways of plant hormones and can improve the resistance of plants to stressors, such as drought, salinity, cold and heavy metals. In addition, hydrogen could delay postharvest ripening and senescence of fruits. Observational evidence has also shown that hydrogen can regulate the flowering time of plants. These results indicate that hydrogen may have great potential applications within agricultural production, indicating that there may be a new ‘hydrogen agricultural era’ to come.

## Introduction

Hydrogen is the most widely distributed element in the world, accounting for more than 75% of the mass of the universe, and it is also the most abound element of human body composition. Hydrogen gas is colorless, odorless and tasteless, and was considered to be physiologically inert molecule, regarding as a potential resource for clean energy in future.

From 1930s to 1940s, some of the bacteria and algae were found capable of producing hydrogen [1,2]. After going through more than half a century, gain little application did the industrialization of hydrogen production by bacteria and algae. But in 2007, things turned the corner. Scientists from Nippon Medical University published a paper about medical protective effect of hydrogen on Nature Medicine which completely updated our knowledge about hydrogen in biology-hydrogen can not only be considered as a source of energy, but also has an therapeutic potent in disease [3]. In this study, the authors found that the hydrogen protected cerebral ischemia-reperfusion injury by selectively reducing · OH and ONOO^-^ which are two most toxic reactive oxygen species in body. This surprising discovery immediately attracted numerous researchers all over the world, and variously new medical and biological effects of hydrogen have been reported after that. It is never coming to mind that hydrogen which had been implied as respiration gas in diving for its inactive in mammals now seems to become a “wonder drug” in fighting diseases. Some researchers in Japan and China have also developed variety of hydrogen related health products which are warmly pursued by the public. Therefore, many researchers also believed that, with deeper digging, hydrogen may play a major role in promoting human health.

Since hydrogen gradually becomes the most shining star in medicine, health care and cosmetic fields, gracefully waving in agricultural production is also hydrogen. People probably did not expect that hydrogen can be used not only for medical treatment and health care, but also may be widely applied to agricultural production. This may lead us to embrace the coming of “the era of hydrogen agricultural”!

### Hydrogen production in higher plants

Early in nineteenth century, researchers had realized the bacteria and algae could synthesis molecular hydrogen. In 1931, researchers reported the first bacteria enzyme which activates molecular hydrogen [4]. In 1942, photochemical production of hydrogen in algae was firstly found [2]. If most bacteria and algae could produce hydrogen under certain conditions [5], what about higher plants? Can higher plants produce hydrogen either?

In 1947, Boichenko claimed that chloroplasts isolated from algae can release hydrogen. Scientists naturally come to the assumption that higher plants whose leaves also contain chloroplasts may able to produce hydrogen [6]. In the year of 1961, the evidence of higher plants leaves releasing and absorbing hydrogen was demonstrated by Sanadze [7]. In 1964, Renwick and his colleges denoted that many higher plants could release hydrogen and exogenous hydrogen could promote the germination rate of winter rye seed [8]. After that hydrogenase with activity of hydrogen production was isolated by Maione and Gibbs from the chloroplast of Chlamydomonas reinhardtii. They had the hypothesis that hydrogenase should also exist in some higher plants [6]. Then confirming evidences–release of hydrogen and detection of hydrogenase activity from barley roots–published by Torres showed that the higher plants can actually release hydrogen [9]. Since then the study on higher plants for hydrogen production is ignored for a long time. One reason for that probably to get clean energy not for its biological effects was the firstly intention of investigating on hydrogen production. Another reason is that the inconvenience of hydrogen collection compared with collection in bacterial and algae.

### Hydrogen effects on higher plants

The first finding of hydrogen effects on higher plants was in 1964, when Renwick *et al.* found hydrogen treated winter rye seeds germinate more rapidly than control [8]. Unfortunately, scientists have not done further study since then. Hydrogen effects on higher plants have not been followed until health effects of hydrogen are generally concerned. Recently, researchers in China preliminarily studied hydrogen effects on higher plants, the results show that the hydrogen has important regulation effect on plant physiological function, especially plays an important role in plant resistance to abiotic stress. The study shows that hydrogen has an important effect on the mung bean, rice [10] and alfalfa (Medicago sativa) [11] seed germination, and the H_2_ pretreatment can improve the rice and Arabidopsis salt stress resistance [12].

Researchers at the Nanjing Agricultural University found that H_2_ pretreatment can induce the expression of heme oxygenase (HO-1) gene, one of Alfalfa antioxidase gene, and enhance its enzyme activity, reducing the oxidative damage caused by paraquat [11]. They presumed that H_2_ might function as an important gaseous molecule that alleviates oxidative stress via HO-1 signalling. They also found that the H_2_ pretreatment can improve salt tolerance in rice and Arabidopsis, and the improvement of salt tolerance may be related to the reduction of reactive oxygen species (ROS) injuries [12]. In addition, they found that hydrogen enhances the resistance of alfalfa to cadmium and aluminum due to the improvement of alfalfa antioxidant capacity induced by hydrogen [13,14].

Researchers at the Southern China Botanical Garden, Chinese Academy of Sciences, and Second Military Medical University in Shanghai confirmed the antioxidant role of hydrogen in rice seedlings, and found that antioxidant enzyme gene expression was induced by H_2_. In addition, upregulation of several phytohormone receptor genes and genes that encode a few key factors involved in plant signaling pathways was detected in rice seedlings treated with hydrogen water. H_2_ production was found to be induced by abscise acid, ethylene, and jasmonate acid, salt, and drought stress and was consistent with hydrogenase activity and the expression of putative hydrogenase genes in rice seedlings. The study suggests that hydrogen might be an important plant gaseous signaling molecules, which may participate in the regulation of plant hormone signaling pathways involved in plant growth and stress adaptation [10].

### “Hydrogen agricultural era” is waving to us

A major feature of modern agriculture is the extensive use of fertilizers and pesticides. Now, the abuse of pesticides and fertilizers causes serious environmental pollution, soil degradation and food safety issues. Due to the safety of H_2_, the convenience and economy of hydrogen water usage, the prospect of its application in agricultural production will be very attractive. Recently, some field trials done by several agricultural research institutions in China shows that hydrogen and hydrogen water seems to be valuable for agricultural production especially for soilless cultivation of crops, and may also have a positive effect on the nutritional value of crops. In the future, farmers may use hydrogen water to replace or partially substitute for pesticide and fertilizer to enhance crop resistance to disease, insect, drought and salinity stress, and improve product quality, increase the yield. How exciting the “hydrogen agricultural era” is! The application of hydrogen in agricultural production may be in the following aspects:

#### Seed germination

Studies show that H_2_ can promote the seed germination rate of winter rye and alfalfa [8]. This finding may promote the application of hydrogen in improving the seed germination rate of plants.

#### Regulation of flowering time

It has been observed that roses and other plants change flowering time after treatment of hydrogen water. It was also found that hydrogen can regulate the expression of plant blossom related plant hormone receptor protein gene [10]. This finding suggests that hydrogen water will have broad application prospects in horticulture.

#### Improvement of crop stress resistance

Drought and salinity stresses often result in crop yield reduction and even death. Studies found that hydrogen water can improve the resistance ability of rice, *Arabidopsis* and *Medicago sativa* plants to salinity, drought and other stresses [11,12]. The crops irrigation or sprinkler irrigation using hydrogen water, will improve the stress resistance of crops, to achieve the purpose of disaster prevention and reduction.

#### Improvement of crop resistance to disease and pests

The study have found that hydrogen can regulate the expression of receptor protein genes of many plant hormone, including some plant hormones associated with disease resistance, such as salicylic acid and jasmonic acid [10]. Irrigation of crops by the use of hydrogen water will likely improve crop resistance to pest and disease leading to substitute for pesticides or reduce the use of pesticides thus it protect environment and improve food security.

#### Improvement of the quality of agricultural products

Hydrogen water irrigation of crops, such as vegetables and fruits, might make them much more delicious.

#### Reducing fertilizer use

H_2_ can regulate the effects of plant hormones such as auxin, cytokine. Hydrogen water treatment can promote the growth of the plant. It has been observed that hydrogen water has a significant effect on the growth of mung bean plants [10]. Therefore, in the future, hydrogen water may be attractively used to irrigate crops, promoting plant growth, and reducing the use of chemical fertilizers.

#### Crop products preservation

The study has been shown that hydrogen water treatment could delay postharvest ripening and senescence of kiwifruit. Reduction of oxidative damage was considered be one of the main mechanisms by which the hydrogen water treatment delays senescence and inhibits respiration of kiwifruit [15]. Owing to the antioxidant properties of hydrogen, hydrogen or hydrogen gas mixtures with other gases may contribute to the preservation of agricultural products. Due to the security of hydrogen, no poison, no residue, it has a strong advantage of food safety compared with other chemical treatment of fresh agricultural products.

“Hydrogen agricultural era” is desirable, but it still requires amounts of deep research and development, which firstly should be to study the mechanism of hydrogen effects on higher plants, to lay a solid theoretical foundation for the application of hydrogen agriculture; and secondly be to do a large scale field experiment, to figure out the precise methods of hydrogen or hydrogen water application in the agricultural production. We believe that, with these problems being solved gradually, “hydrogen agricultural era” will step to us.

## Competing interest

We are here to formly state that all the authors have no competing interest on this article.

## Authors’ contribution

JZ participated in conception, designing and writing the article. ZY and XS contributed to the critical review and revision of the manuscript. All authors have seen and approved the final version of the manuscript.
